# Isotopic Evidence of a Wide Spectrum of Feeding Strategies in Southern Hemisphere Humpback Whale Baleen Records

**DOI:** 10.1371/journal.pone.0156698

**Published:** 2016-05-31

**Authors:** Pascale Eisenmann, Brian Fry, Carly Holyoake, Douglas Coughran, Steve Nicol, Susan Bengtson Nash

**Affiliations:** 1 Environmental Futures Research Institute, Griffith University, Brisbane QLD 4111, Australia; 2 Australian Rivers Institute, Griffith University, Brisbane QLD 4111, Australia; 3 Murdoch University, Perth WA 6150, Australia; 4 Department of Parks and Wildlife, Kensington WA 6151, Australia; 5 Institute for Marine and Antarctic Studies, University of Tasmania, Hobart TAS 7000, Australia; UC Santa Cruz Department of Ecology and Evolutionary Biology, UNITED STATES

## Abstract

Our current understanding of Southern hemisphere humpback whale (*Megaptera novaeangliae*) ecology assumes high-fidelity feeding on Antarctic krill in Antarctic waters during summer, followed by fasting during their annual migration to and from equatorial breeding grounds. An increase in the number of reported departures from this feeding/fasting model suggests that the current model may be oversimplified or, alternatively, undergoing contemporary change. Information about the feeding and fasting cycles of the two Australian breeding populations of humpback whales were obtained through stable isotope analysis of baleen plates from stranded adult individuals. Comparison of isotope profiles showed that individuals from the West Australian breeding population strongly adhered to the classical feeding model. By contrast, East Australian population individuals demonstrated greater heterogeneity in their feeding. On a spectrum from exclusive Antarctic feeding to exclusive feeding in temperate waters, three different strategies were assigned and discussed: classical feeders, supplemental feeders, and temperate zone feeders. Diversity in the inter-annual feeding strategies of humpback whales demonstrates the feeding plasticity of the species, but could also be indicative of changing dynamics within the Antarctic sea-ice ecosystem. This study presents the first investigation of trophodynamics in Southern hemisphere humpback whales derived from baleen plates, and further provides the first estimates of baleen plate elongation rates in the species.

## Introduction

Southern hemisphere humpback whales (SHHW) exhibit one of the longest mammalian migration on Earth, travelling close to 10 000 kilometres between their equatorial breeding grounds and Antarctic feeding grounds [1–3]. The classical feeding ecology model for these populations assumes high-fidelity summer feeding on Antarctic krill (*Euphausia superba*) followed by fasting throughout the entire migration, with feeding only resumed in Antarctic waters the following summer [4–7]. Because they are capital breeders, the humpback whales breed, calve and nurse during the migratory fast. As such, the SHHWs rely on the presence of high krill biomass to feed, and to replenish their blubber stores for a successful migration and reproduction.

**Fig 1:**
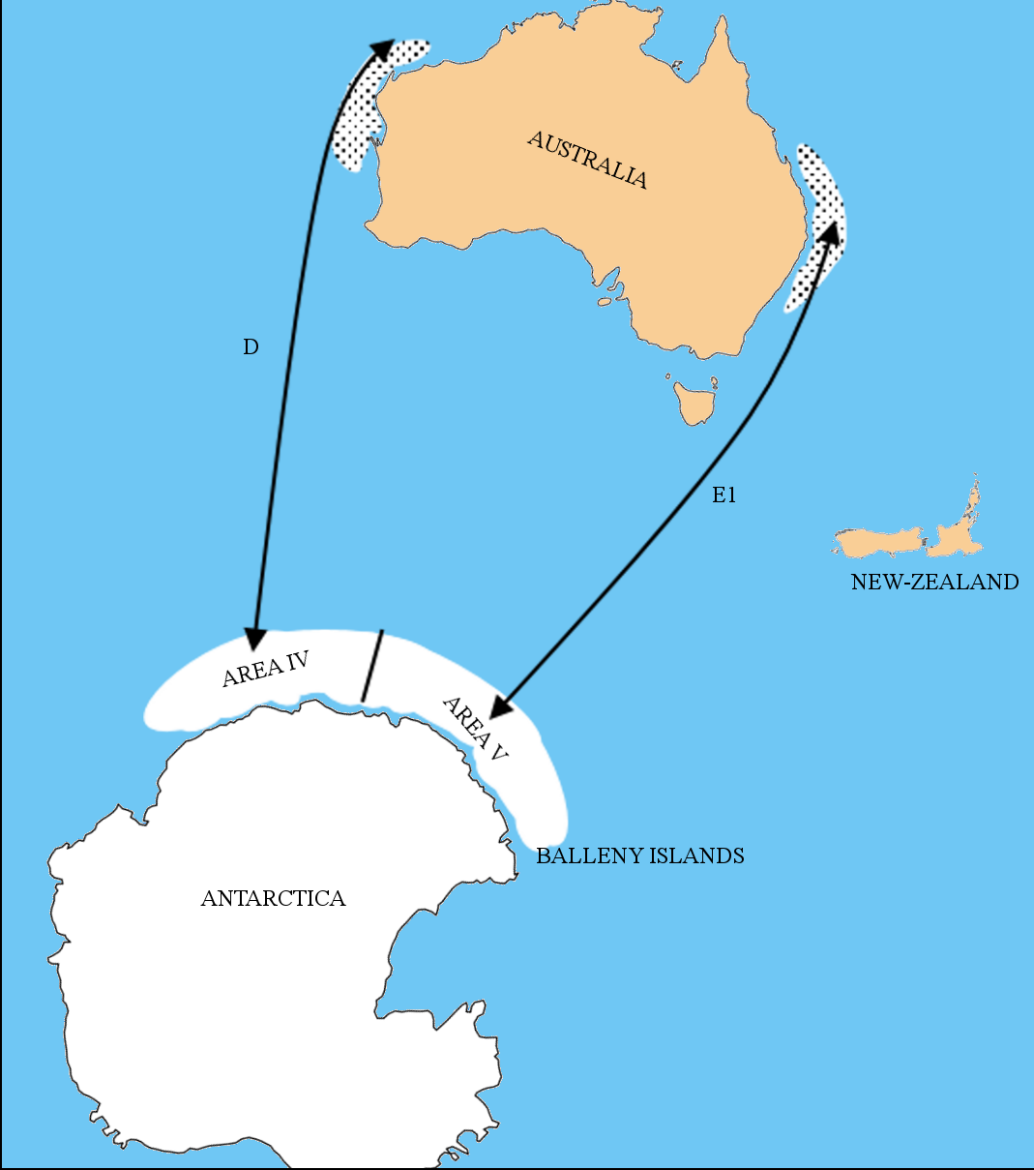
Migration, feeding grounds and breeding grounds of the Australian humpback whale populations. Approximate feeding grounds of the D and E1 populations are the historical stock boundaries Area IV and V, as defined by the International Whaling Commission. Approximate breeding grounds are represented by the dotted areas, and migration routes are indicated by arrows.

There are 7 distinct breeding stocks (A-G) of SHHW recognised by the International Whaling Commission (IWC) [8]. The populations migrating along the western and eastern Australian coasts are classified as the D and E1 breeding stocks, respectively (Fig 1) [8]. While uncertainty still surrounds the extent and inter-annual consistency of the Antarctic feeding grounds associated with both of these populations, mark-recapture data evidence led the IWC to allocate feeding areas IV and V (Fig 1) to the D and E1 breeding stocks respectively. Tagging evidence corroborates the significance of area V, which include the Balleny Islands, as a summer feeding ground for the E1 population [9, 10].

The narrow feeding niche of SHHWs places them at an elevated risk of exposure to the detrimental effects of climate-induced environmental change. In a warming climate, the Antarctic cryosphere is expected to undergo dramatic change [11, 12]. Sea-ice represents a critical nursing ground for Antarctic krill larvae, hence a decline in sea-ice cover and duration has been predicted to have a devastating impact on Antarctic krill biomass [13–15]. One study has predicted that unmitigated CO_2_ emissions will result in a complete collapse of Antarctic krill stocks by the year 2300 [16]. Negative impacts to this keystone species carry repercussions for predators within the Antarctic sea-ice ecosystem, including SHHWs. Adverse impacts to the energetic health of capital breeders such as SHHWs have direct implications for population fecundity, survival, and toxicological risk of lipophilic contaminant burdens [17–22].

In recent years, departures from the classical feeding model have been observed in several SHHW populations, with feeding along their migration routes reported in breeding populations from Western Africa, East Australia, and both Pacific and Atlantic sides of South America [8, 23–29]. A rise in such observations may simply be an artefact of the rapid, post-whaling re-expansion of the Southern Ocean baleen whale stocks [30, 31]. Alternatively, these observations may signal changing dynamics within the Antarctic sea-ice ecosystem [13, 14, 32], or likely, a combination of both. Nonetheless, all scenarios place the classical feeding model of SHHW under scrutiny. New research is required to carefully evaluate whether the classical model is an accurate representation of present day feeding, or in fact an over-simplification of feeding by a highly adaptable species.

Stable isotopes (SI) are regularly used in trophodynamic studies because the isotopic composition of an organism closely reflects the composition of ingested food [33, 34]. Stable isotope measurements of δ^13^C and δ^15^N can show seasonal variations in diet and feeding location [35, 36], as exemplified in studies of migratory marine fauna such as shrimp [37, 38], southern right whales [39] and fin whales [40]. Carbon isotopes are typically used to distinguish two geographically distinct food webs. Altabet and Francois [41] showed that surface water δ^13^C values of particulate organic material are near -22‰ in temperate latitudes but decrease to -25‰ closer to Antarctica. Animals feeding in Antarctic food webs show correspondingly low carbon isotope values [42–45] compared to temperate food webs [46, 47]. Nitrogen isotope values more strongly record trophic level and metabolic changes such as fasting [33, 48, 49]. Several studies have shown that fasting introduces variations in δ^15^N independently of food trophic level, although the variations appear to be species and tissue specific [39, 50–53]. To our knowledge, no studies on humpback whale fasting exist, hence no assumptions were made regarding the possible effects of fasting on baleen δ^15^N values.

Tissues with incremental growth, such as bone, hair or nails retain long-term records of historical feeding patterns [50, 54]. Whale baleen plates are keratinous hair-like structures that grow continuously, although older sections are eventually worn-off. Because keratinous materials are formed from multiple metabolic pools with different turnover times [50, 54], shifts in feeding behaviour are not recorded instantly. The dominant pools in keratin, however, appear to have a fast turnover of one to seven days [50, 54] during which the organism equilibrates with the isotopic values of newly ingested food. Dietary changes have been found to be well resolved in baleen, with the most recently formed section of the plate reflecting the isotopic composition of the diet from the past two weeks [39, 50, 52].

In this study, we investigate the validity of the classical SHHW feeding model through analysis of baleen stable isotope records from twenty stranded adult individuals, with uncertain cause of deaths. We hypothesized that isotopic signals would indicate if and when a change to classical feeding had occurred. This study further estimates elongation rates for baleen plates in SHHW, and provides insight into metabolic patterns associated with the migratory life-history of SHHWs, highlighting isotopic changes associated with fasting.

## Methods

### Ethics statement

The samples were obtained from necropsied whales that stranded on the Australian coast between 1940 and 2015. They were preserved in museum collection until requested for analyses. In the case of the Southern Ocean Persistent Organic Pollutant Program (SOPOPP) collection, the necropsy samples were obtained under Scientific Purposes permit WISP14251214, granted by the QLD Department of Environment and Heritage Protection and permit ENV1710AEC granted by the Griffith University Animal Ethics Committee.

### Sample collation

Baleen plates were collected from SHHW animals stranded between 1940 and 2015 (Table 1, S1 Table) along Australian shores. Young animals smaller than 6.4m in length were excluded from analysis as they were likely dependent on maternal provisioning at the time of death. Preliminary analysis of these younger animals has shown relatively constant isotope values (Eisenmann, unpublished data), as expected if calves fed on maternal reserves. If no size information was recorded at the time of stranding, plates with isotopic oscillations indicative of one or more migrational cycles were considered to be of adults. In total, plates from thirteen E1 population whales and seven D population whales were analysed (Table 1, S1 Table).

**Table 1:** Whales sampled for baleen isotope records.

Name	Date Collected	Sex	Plate length (cm)
E01	1940	/	34
E03	1989	F	30
E05	22/10/98	F	30
E08	07/06/10	/	17
E10	1/10/11	M	63
E12	06/11/11	M	39
E13	07/11/11	M	33
E14	05/06/12	M	22
E18	December 2012	/	26
E23	7/07/2014	/	7
E24	31/08/10	/	46
E26	01/11/10	/	25
E27	10/03/89	M	17
D01	30/07/07	/	42
D10	10/10/13	/	36
D11	16/10/13	F	8
D12	18/08/13	F	62
D13	12/04/14	M	14
D14	12/07/14	/	19
D15	29/07/14	F	33

“E” and “D” are the respective populations. Sex was determined either through visual inspection during sample collection or through DNA-sexing of matching blubber samples (if available).

Baleen plates were collected opportunistically. These plates were of varying length, reflecting their position in the mouth. Population separation of the animals was done according to location of stranding. Baleen plates from the E1 breeding population were obtained from the Museum Victoria, the Australian Museum, the Queensland Museum, the Ceduna Trust museum, the South Australian Museum, and the Southern Ocean Persistent Organic Pollutant Program (SOPOPP) collection at Griffith University (S1 Table). Two plates were further obtained courtesy of the Tasmanian Department of Primary Industries, Parks, Water and Environment. Baleen plates from the D breeding population were provided by Dr. Carly Holyoake and by Mr. Douglas Coughran (S1 Table).

### Stable Isotope Analysis

Baleen plates were first washed to remove sand, blood and other particulates that may have accumulated during collection, transportation and storage. The first wash consisted of deionized water and scrubbing with a steel brush to remove coarser contaminants. Complete removal of tissue such as the gum was done along the external edge to allow full access of the emerging baleen hair. The plate was then washed a second time in a 2:1 chloroform:methanol solution to remove any oils and lipids [52, 55]. Plates were oven-dried at 58°C to constant weight. On average, baleen plates stayed in the chloroform:methanol wash for 1 day, followed by 2 days in the drying oven. Those times varied somewhat depending on baleen plate size and collection date.

SIA required 1–2 mg of clean sample material. In the case of keratin-composed baleen plates, using an engraving tool for sampling was sufficient to create enough powder or slivers for analysis. Samples were taken every centimetre along the outer edge of the plate. The outer edge was preferable as the keratin filaments were unbroken and the edge was smooth compared to the inner side, where keratin filaments were loose to provide a “netting” network used to retain prey. Measurements started at the oldest point on the baleen (0 cm), and continued until under the gum line (newest part of the baleen). Powder or a sliver of baleen was weighed into tin capsules for isotope analysis [55].

All stable isotope abundances are calculated in ‰ using the following formula:

$$\delta X=\left[\left(\frac{Rsample}{Rstandard}\right)-1\right]\;x\;1000$$ (1)

where X = ^13^C or ^15^N, and R = the respective ratio ^13^C/^12^C or ^15^N/^14^N. The international reference standards for carbon and nitrogen are respectively Vienna Pee Dee Belemnite and N_2_ in air. International standards IAEA-CH_6_ for carbon and IAEA N1 for nitrogen were used for calibration of laboratory standards sucrose and (NH_4_)_2_SO_4_. The preparation system was a Europa EA-GSL interfaced to a SERCON Hydra 20–20 isotope ratio mass-spectrometer (IRMS). Based on analysis of replicate standards, the standard deviation for δ^13^C and δ^15^N respectively averaged 0.1‰ and 0.15‰.

### Source prediction and trophic fractionation

Measured baleen isotope values were compared to literature-derived estimates of three distinct prey sources (combining prey type and feeding location). The three estimates are presented in Table 2 together with corresponding references [42–47]. Literature averages represent data accumulated across a range of years, so that temporal variability was at least partially accounted for. We included the standard deviation of all values in the defined prey isotope ranges to account for possible taxonomic and sub-regional variations in δ^13^C and δ^15^N (Table 2).

**Table 2:** Reported isotopic values for possible humpback whale prey items.

	δ^13^C	SD	δ^15^N	SD	Literature
**Antarctic Krill (*Euphausia superba*)**	-27.1	1.74	3.2	1.69	Wada, Terazaki [42], Cherel [43], Hall-Aspland, Hall [44], Hodum and Hobson [45]
**Australian Krill (averaged)**	-19.7	0.40	8.3	0.50	Harris, Young [47]
**Australian pelagic fish (averaged)**	-18.7	0.85	11.8	0.74	Calculated from Davenport and Bax [46]

Antarctic krill samples originate from within Area IV and V and were collected between 1982 and 2002. Multiple species of Australian krill were collected off the East Australian coast in 2010, and multiple species of Australian pelagic fishes (both secondary and tertiary consumers) were sampled in the Bass straits, off the South-east Australian coast, between 1993 and 1996. Australian prey items were averaged, because any of the species potentially could be a prey item.

In all cases, literature-derived prey estimates were adjusted to account for trophic fractionation and represent the baleen isotopic values of whales consuming these prey (S2 Table). The adjusted estimates are calculated as follow:

$$\delta X_{B}={TF}_{x}+\;\delta X_{P}\pm {\text{SD}}_{P}$$ (2)

**Fig 2:**
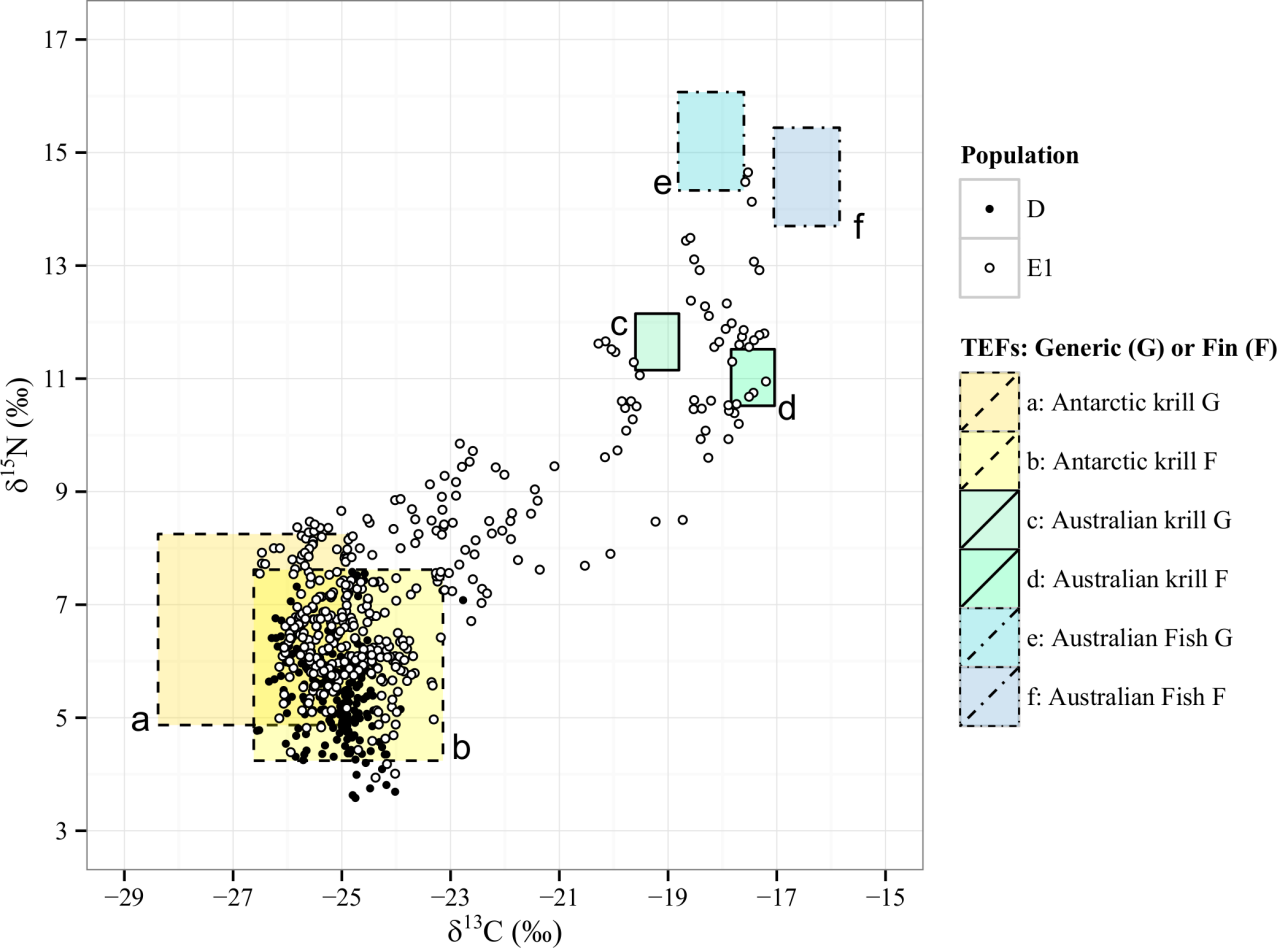
Bivariate comparison of whale and prey isotope values corrected using two sets of TFs. Each prey source is plotted twice depending on the set of TFs: rectangles a, c, and e represent the generic set of TFs: + 3.4‰ for δ^15^N and + 0.5‰ for δ^13^C, while rectangles b, d, and f represent the fin whale-specific set of TFs: +2.77‰ for δ^15^N and +2.26‰ for δ^13^C. Sources: Antarctic Krill (dashed line), Australian Krill (full line), average Australian fish species (dash-dot line). Each zone is therefore created using isotopic coordinates consistent with prey values and corrected for trophic fractionation. The whale isotope data should plot within the zone appropriate to the prey consumed by the individual.

Where δX = δ^13^C or δ^15^N values, B is baleen, P is prey, X = ^13^C or ^15^N, TF is the trophic enrichment factor, and SD is the standard deviation. Two sets of TF estimates were initially compared (S2 Table, Fig 2). The first set corresponds to generic estimates of +3.4‰ for nitrogen and 0.5‰ for carbon. Although δ^13^C TEF values are known to range from 0.5–1.5‰ [48, 56], the generic estimates given here are the most commonly used for species with unknown TFs. Because a recent study on krill-feeding northern hemisphere fin whales reported TFs of +2.77‰ for nitrogen isotopes and +2.26‰ for carbon isotopes in baleen plates [57], this TF set was also included in the preliminary analysis (Fig 2).

The isotopic values from the known diet of SH humpback whales aligned more closely with fin whale-specific TF estimates (+2.77‰ per trophic level for nitrogen and +2.26‰ for carbon) than generic estimates (+3.4‰ per trophic level for nitrogen and +0.5‰ for carbon; Fig 2). The fin whale specific TF set was thus used in our interpretations. As the estimates do not overlap in δ^13^C or δ^15^N, they provide distinct isotopic zones specific to the consumption of that prey (Fig 2).

### Statistical analyses

Statistical analyses were performed using SPSS for Windows. The whole dataset, comprising each individual observation from every baleen, was tested for normality using a Shapiro-Wilk test and equality of variances using a non-parametric Levene test. Because the distributions and variances for δ^13^C and δ^15^N across the populations were not normal (p<0.05), we used a Kruskal-Wallis non-parametric comparison of ranked means, followed by appropriate post-hoc tests to look for differences between feeding categories and between populations.

Feeding categories were assigned according to adherence of the observed individual isotopic profiles to the three distinct feeding estimates.

## Results and Discussion

### Baleen elongation rates, and annual isotopic features

It has previously been reported that baleen plate isotope records were consistent between plates within a single individual in bowhead whales [58, 59], and gray whales [60]. To confirm that SHHW plates contain the same isotopic patterns and grow at the same rate regardless of mouth location, we analysed paired plates from whales E14 and D01 (S1 Fig). There were minimal isotopic differences between the plates for each individual, with the main difference due to the length of the plates: a shorter plate covered a smaller temporal record than a longer one (D01, S1 Fig). This observation would suggest that the size differences between plates of a single individual are due to the rate at which there are eroded during feeding and not due to variations in elongation rate, as found in other cetacean species.

Recurring δ^15^N minima (e.g. S1 Fig, arrows) were visible in plates longer than 15cm. These minima were attributed to annual Antarctic summer feeding as justified by three separate lines of evidence. Firstly, the δ^15^N values for Antarctic krill were the lowest values of all the possible prey items identified (Table 2). It then follows that whales feeding on Antarctic krill during the summer would have low δ^15^N values in comparison to whales that fed on other prey. Secondly, SHHWs spend only the most productive months (November to March) feeding in Antarctica. By extension, the comparatively shorter periods in δ^15^N values could therefore be attributed to the intensive feeding periods with the longer periods of elevated isotope values being attributed to the extended fast. Finally, most of the whales show locally higher δ^15^N values in the most recently formed part of the baleen (closest to stranding time, e.g.: S1 Fig, D01 cm #40–42). As sampled individuals were collected in Australian waters, higher δ^15^N values can confidently be associated with migration and fasting in whales with low Antarctic δ^13^C, or in Australian feeding with high δ^13^C, such as the temperate zone feeders discussed further below.

Overall, the observed general annual pattern was that lower δ^15^N values were associated with Antarctic feeding, and moderately higher plateauing δ^15^N values were associated with fasting during the migration. These δ^15^N cycles were generally regular and interpreted as annual, in line with previous studies [39, 51, 52, 55, 58, 61]. With this understanding of the annual nature of δ^15^N in SHHWs, the length of baleen between two δ^15^N minima was used to estimate growth occurring in one year. The average adult humpback whale plate contained three years of data, although two plates from different individuals were in excess of 60 cm in length, corresponding to approximately five years of migration cycles. Both of these individuals were large, likely fully grown, animals (14 m and 17 m in length respectively) and had long baleen plates (Table 1). The baleen elongation rates for adult humpback baleen observed in this study varied from 12–20 cm per year, with rates averaging 16 cm/yr and decreasing to 12 cm/yr in larger and presumably older animals. These adult elongation rates are similar to those of southern right whales [39], fin whales [51] and gray whales [52], that have been shown to average 12–24 cm per year for adults.

The annual oscillations in δ^15^N likely indicate the alternating use of two differing nitrogen pools, or the use of two different nitrogen metabolism. Two or more isotopically distinct nitrogen pools have been identified in mammals [54], birds [62] and in marine animals such as shrimp and salmon [63, 64], and there is increasing evidence for multiple types of nitrogen metabolism affecting the isotopic values of marine animals [65, 66]. For SHHWs, the δ^15^N minima likely marks the end of summer Antarctic feeding, during which direct dietary input has supported whale growth and formation of blubber reserves. At the start of the migratory fast, δ^15^N values rise as whales switch nitrogen pool or metabolism. While the principal cost of fasting is met by the accumulated energy reserves of the blubber triglycerides, lipids lack nitrogen, and a non-critical protein source is required for tissue maintenance and regeneration. Although not yet explicitly identified, this second, non-essential protein pool is used during the normal migratory fast. Use of this pool results in the observed high δ^15^N plateau after the summer minimum. The decline in δ^15^N at the end of the plateau marks the resumption of Antarctic feeding.

### Heterogeneity in individual feeding records

The baleen isotope records evaluated in this study did not support the null hypothesis that all SHHWs adhere to a high-fidelity Antarctic krill diet interspersed with extended periods of fasting. Instead, individual feeding records fell on a spectrum from those following the classical krill-feeding/fasting model to those shifting to permanent feeding within temperate waters. The three feeding categories, based upon literature-derived prey choices, were termed: 1) Classical feeders, 2) Supplementary feeders, and 3) Temperate zone feeders. Individuals were assigned a feeding category according to visual examination of their isotope profiles, and comparison to the prey consumption zone estimates (S2 Table, Table 3).

**Table 3:** Means, SD, minima/maxima for δ^13^C and δ^15^N for all whales.

Feeding category	ID	n	Mean ± SD δ^13^C (‰)	Min. δ^13^C (‰)	Max. δ^13^C (‰)	Mean ± SD δ^15^N (‰)	Min. δ^15^N (‰)	Max. δ^15^N (‰)
**Classical D**	D01	43	-25.1 ± 0.4	-26.1	-24.6	5.5 ± 0.5	4.4	6.1
	D11	9	-25.4 ± 0.3	-25.7	-24.8	5.9 ± 0.1	5.7	6.1
	D12	63	-24.7 ± 0.4	-25.7	-23.9	4.9 ± 0.7	3.6	6.3
	D13	15	-26.0 ± 0.1	-26.3	-25.8	6.5 ± 0.3	6.1	7.3
	D14	20	-25.9 ± 0.4	-26.6	-25.3	5.4 ± 0.8	4.3	6.6
	D15	34	-25.2 ± 0.4	-25.9	-24.4	5.7 ± 0.6	4.3	6.5
	Total	184	-25.1 ± 0.6	-26.6	-23.9	5.4 ± 0.7	3.6	7.3
**Classical E1**	E08	18	-25.7 ± 0.4	-26.1	-24.9	6.4 ± 0.2	6.0	6.8
	E10	64	-24.3 ± 0.5	-25.7	-23.3	5.6 ± 0.6	4.0	6.6
	E24	46	-25.2 ± 0.4	-26.0	-24.5	6.3 ± 0.7	4.4	7.2
	E26	26	-25.3 ± 0.5	-26.2	-24.3	6.1 ± 0.7	5.0	7.4
	Total	154	-24.9 ± 0.7	-26.2	-23.3	6.0 ± 0.7	4.0	7.4
**Supplementary**	D10	37	-24.9 ± 0.6	-25.4	-22.8	6.2 ± 0.9	4.8	7.6
	E03	31	-24.0 ± 0.9	-25.1	-22.3	6.7 ± 0.6	5.7	7.9
	E12	40	-25.3 ± 0.7	-26.1	-23.2	7.6 ± 0.8	5.9	8.9
	E13	34	-24.7 ± 0.4	-25.4	-23.0	7.3 ± 0.8	5.8	9.1
	E14	23	-25.2 ± 1.3	-26.5	-23.0	7.2 ± 1.1	5.4	8.5
	E23	8	-25.7 ± 0.2	-25.9	-25.4	7.5 ± 0.4	6.8	7.9
	E27	18	-24.9 ± 1.2	-25.9	-22.0	7.8 ± 1.0	6.7	9.3
	Total	191	-24.9 ± 0.9	-26.5	-22.0	7.1 ± 1.0	4.8	9.3
**Temperate zone**	E01	35	-20.9 ± 2.0	-23.3	-17.8	8.7 ± 1.1	7.2	10.6
	E05	31	-17.8 ± 0.4	-18.7	-17.2	12.1 ± 1.2	10.2	14.7
	E18	27	-21.6 ± 2.0	-25.1	-19.5	9.9 ± 1.2	7.6	11.7
	Total	93	-20.1 ± 2.3	-25.1	-17.2	10.2 ± 1.9	7.2	14.7
**All individuals**	Total	657	-24.2 ± 2.1	-26.6	-17.2	6.8 ± 1.9	3.6	14.7

We report the means, standard deviations, and minimum/maximum isotope values for each individual as well as assigned feeding categories in Table 3.

#### Classical feeders

Classical feeders were defined as individuals with isotopic profiles consistent with continual provisioning (dietary provenance) from a low trophic level, Antarctic prey source. The literature-derived estimates of the isotopic range representing Antarctic krill feeding were calculated to account for possible temporal and geographical variations reflected in δ^13^C and δ^15^N. More localised feeding grounds with distinctive isotope values may exist within this broad Antarctic range. Typical classical feeder isotopic profiles are exemplified by annual oscillations in δ^15^N and stable δ^13^C values, both confined within the Antarctic range estimates (S2 Fig).

**Fig 3:**
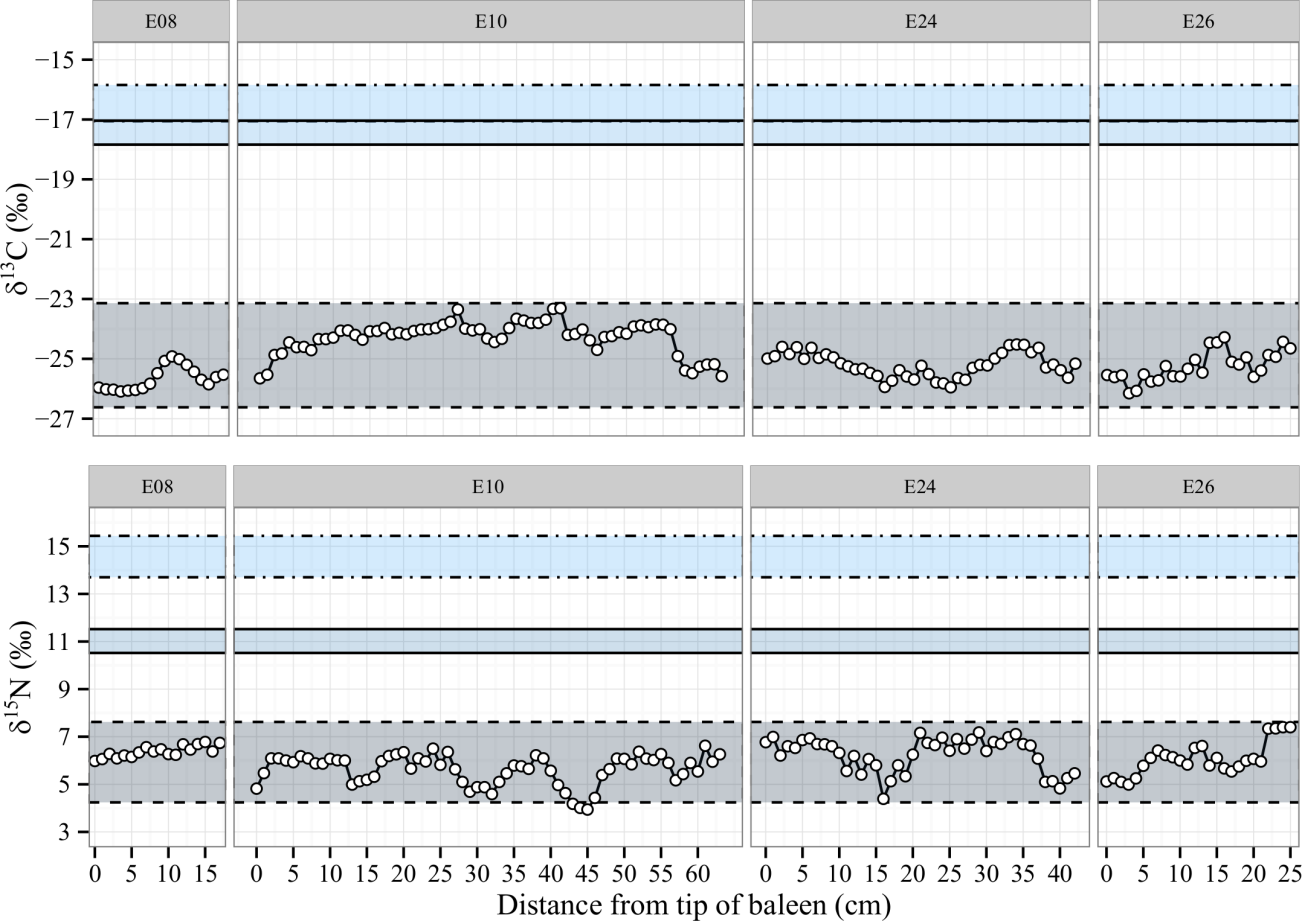
Isotope profiles for E1 population classical feeders. Each horizontal zone is created using isotopic coordinates consistent with prey values and corrected for trophic fractionation. The whale isotope data plots within the zone appropriate to the prey consumed by the individual. Food zones: Antarctic Krill (dashed line), Australian Krill (full line), average Australian fish species (dotted line). Time flows from left to right.

**Fig 4:**
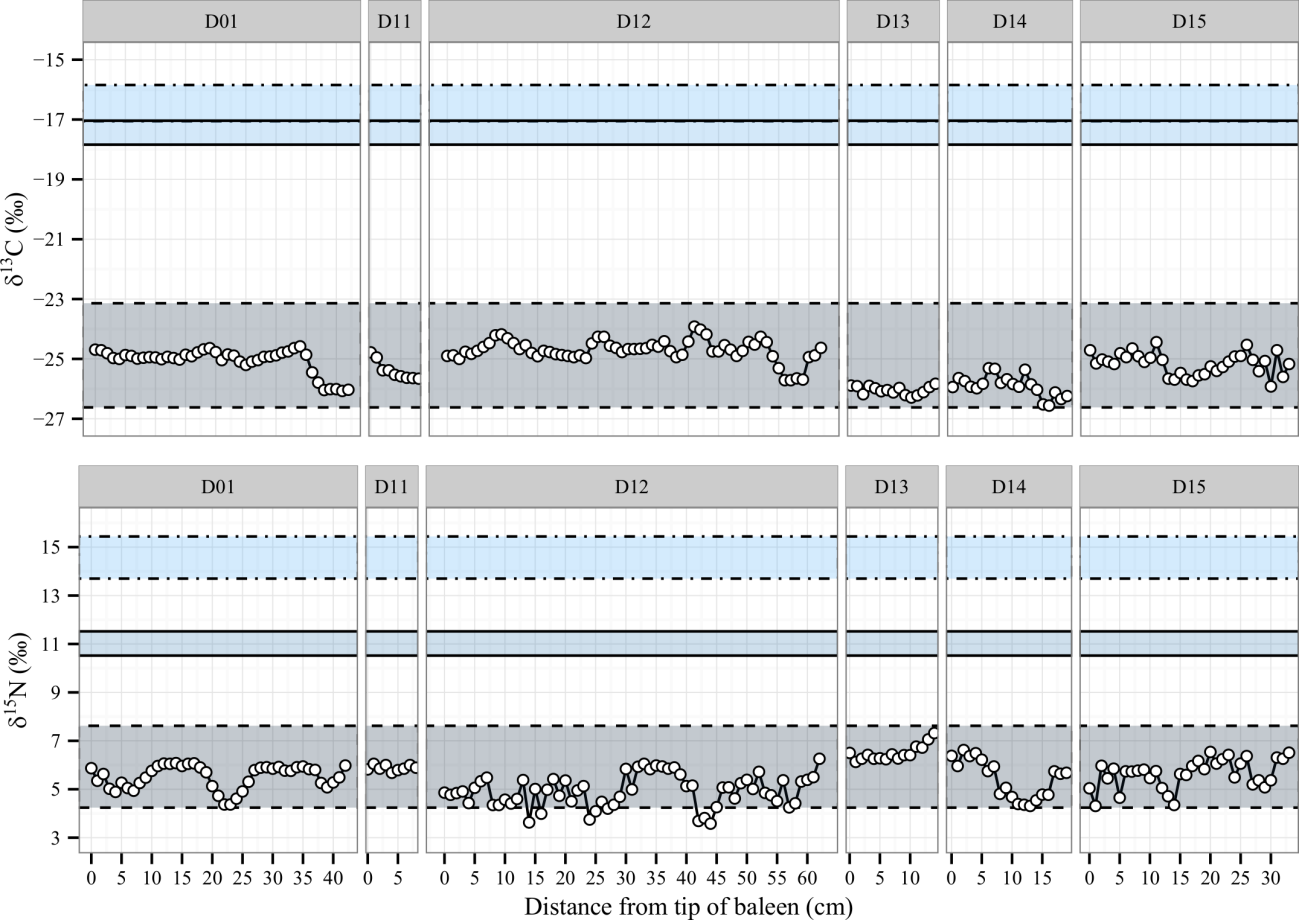
Isotope profiles for D population classical feeders. Each horizontal zone is created using isotopic coordinates consistent with prey values and corrected for trophic fractionation. The whale isotope data plots within the zone appropriate to the prey consumed by the individual. Food zones: Antarctic Krill (dashed line), Australian Krill (full line), average Australian fish species (dotted line). Time flows from left to right.

Among classical feeders of the E1 population, localised carbon maxima and minima were visible in E08 (Fig 3, cm #7–15), E24 (Fig 3, cm #15–25) and E26 (Fig 3, cm #13–16), as well as a slow shift from -26‰ to -23‰ and back down to -26‰ over the course of 4 years in whale E10. Similar examples of carbon anomalies in the classical feeders from D population can be seen in D01 (Fig 4, cm #35–42), D12 (Fig 4, cm #5–10, #23–28, #40–44 and #50–63).

An overall rise in δ^15^N unmatched in δ^13^C were visible in E10 (Fig 3, cm #45–63), E24 (Fig 3, cm #15–25) and E26 (Fig 3, cm #0–10), as well as D01 (Fig 4, cm #40–42) and D13 (Fig 4, cm #10–13). Increases in δ^15^N not paired with increases in δ^13^C may be representative of metabolic changes during fasting when the body shifts from diet-derived pools to body stores [67, 68]. In contrast, the δ^13^C remains the same, as the geographical location of the original food web from which body reserves were created stays the same. Another explanation for such increases in δ^15^N decoupled from δ^13^C could be a shift to a higher trophic level of Antarctic feeding, either by the krill or the whales, but in insufficient quantities to exit the Antarctic δ^13^C isotope range based on average krill-feeding values.

Unfortunately, plates for whales E08, D11, D13 and D14 were quite short and close to our estimated one-year growth, complicating the identification of annual variations consistent with migration. Finally, the comparative shortness of the last nitrogen isotope minima in whales E10 (Fig 3, cm #37–42) and D01 (Fig 4, cm #55–41) may be caused by a shorter migration period. This would imply that the whales spent less time that year feeding within Antarctic waters before returning to Australia. Such a scenario would result in a lower volume of accumulated energy stores. Other possibilities should not be dismissed however, such as feeding on atypical prey with different nitrogen composition than body reserves [42, 69]. Compound specific isotope analysis [49, 65, 70–72] may help resolve these possible trophic-level shifts in future studies of baleen.

#### Supplementary feeders

SHHW supplementary feeders are defined as animals that diverged slightly from the classical feeding model, as demonstrated by carbon and nitrogen isotope values occasionally exceeding the Antarctic krill-feeding range (S3 Fig). Principal provisioning could still be attributed to an Antarctic, low trophic level food source because the higher δ^13^C and δ^15^N values never reached the temperate water feeding ranges (S3 Fig). The comparatively higher δ^13^C and δ^15^N values instead displayed intermediate values between the Antarctic and Australian isotope ranges, and were therefore attributed to supplementary feeding.

**Fig 5:**
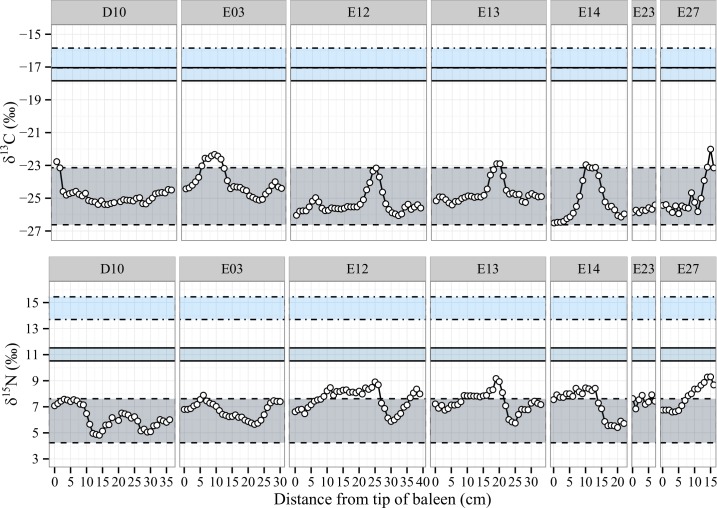
Isotope profiles of supplementary feeders from both populations. Each horizontal zone is created using isotopic coordinates consistent with prey values and corrected for trophic fractionation. The whale isotope data plots within the zone appropriate to the prey consumed by the individual. Food zones: Antarctic Krill (dashed line), Australian Krill (full line), average Australian fish species (dotted line). Time flows from left to right.

Supplementary feeding can be split into two different behaviours, which result in slightly different isotope profiles. The first behaviour is supplementary feeding at a higher trophic level than krill (such as fish) but within Antarctica. This results in δ^13^C values within the defined carbon Antarctic range and increased δ^15^N values possibly outside the Antarctic range. Baleen profiles including this kind of supplementary feeding are E12 (Fig 5, cm #9–20), E13 (Fig 5, cm #0–15), E23 (Fig 5, cm #0–7) and E27 (Fig 5, cm #0–10).

The second behaviour corresponds to discrete feeding events in temperate zone waters during the migration, causing isotope values to increase for both δ^13^C and δ^15^N at the same time. This occurs in whales D10 (Fig 5, cm #0–3), E03 (Fig 5, cm #5–10), E12 (Fig 5, cm #22–29), E13 (Fig 5, cm #15–22), E14 (Fig 5, cm #8–14) and E27 (Fig 5, cm #11–16), Because energy intake during migration is likely to represent only a fraction of the annual requirements, isotopic composition of the baleen shifts only slightly and may not turn-over completely to temperate zone values.

#### Temperate zone feeders

**Fig 6:**
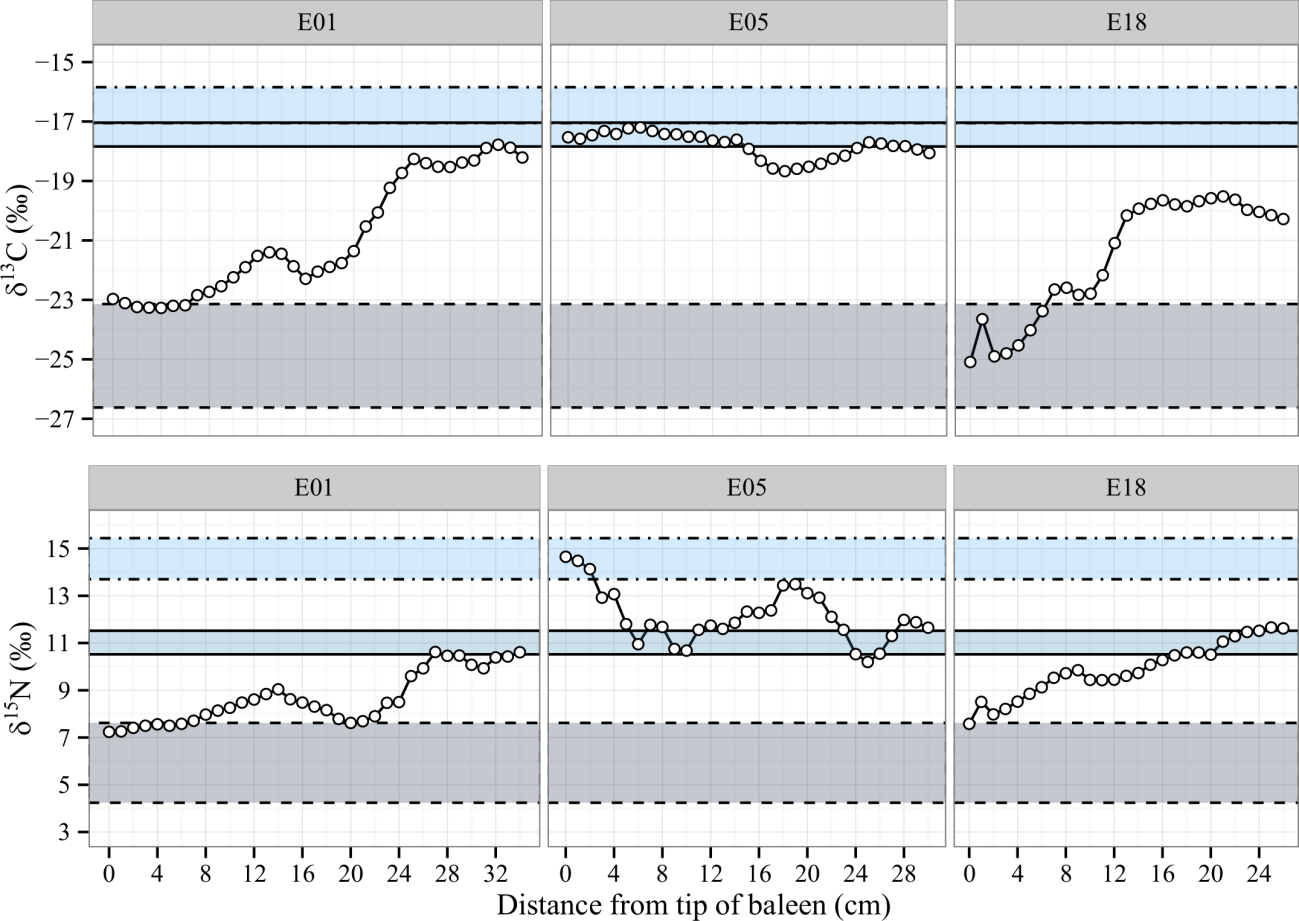
Isotope profiles for temperate zone feeders. Each horizontal zone is created using isotopic coordinates consistent with prey values and corrected for trophic fractionation. The whale isotope data plots within the zone appropriate to the prey consumed by the individual. Food zones: Antarctic Krill (dashed line), Australian Krill (full line), average Australian fish species (dotted line). Time flows from left to right.

The final category of whales, termed temperate zone feeders, are animals that undertook significant feeding outside of the Antarctic (S4 Fig). Two of the individuals exhibited a slow turnover from Antarctic feeding to Australian feeding (E01 and E18, Fig 6) while the isotopic record from the third individual did not contain any remnants of Antarctic provisioning (E05, Fig 6). These individuals showed the most surprising isotopic signals of the entire sample set with animals seemingly forgoing Antarctic feeding entirely, and possibly remaining most of the year prior to death in Australian waters, feeding on Australian prey.

Unlike the animals discussed earlier, E01 and E18 (Fig 6) show no large cyclic variations indicative of a seasonal shift in prey or feeding location. Instead, there is a gradual directional shift from the Antarctic feeding range to temperate prey (Fig 6). Because both E01 and E18 stranded in southern Victoria, it is possible that the year preceding death was spent around Tasmania instead of returning to Antarctic feeding grounds. Accounts of partial migration [73] in SHHWs populations exist [8, 74], so it is possible that these two whales are Australian examples of non- or partially migrating whales. This is further supported in whale E18 by its stranding record. Although the exact day is unclear, stranding occurred during the austral summer in Victoria (S1 Table), when the whales are expected to be in Antarctic waters replenishing their blubber stores.

E05 (Fig 6) has δ^15^N and δ^13^C signals reminiscent of the classical feeding cycles (Figs 3 and 4), but these signals are displaced into the Australian feeding zone. It is possible that this animal is a very extreme example of partial migration, with the baleen record no longer showing any Antarctic signal. Furthermore, the δ^15^N and δ^13^C values from whale E05 rise and fall at opposite times. This could be explained if this whale continued the feeding /fasting regime, but never travelled all the way to the Antarctic feeding grounds, instead remaining within Australian waters and feeding on Australian krill species.

It is important to acknowledge that supplementary feeding and partial migrations may not be recent behavioural changes in SHHWs, nor indeed the behaviour of healthy individuals. The 1940 collection date for E01 and the departure from the classical feeding model apparent in its isotope profile (Fig 6) provide evidence of historical feeding outside of Antarctica.

Changes in ocean biochemistry may also be reflected with the 1940 E01 profile. Studies have shown that ocean acidification [69, 75] and low levels of bio-available iron [76] may cause a decrease in δ^13^C values in the lowest trophic levels of the food web over time [69]. Because anthropogenic releases of CO_2_ in the atmosphere have been shown to contribute to ocean acidification [69, 75], and the possible effects of intensive whaling on iron fertilisation are being brought to light [76], a decrease in the carbon isotope baseline between 1940 and modern Antarctica is possible. This change would not be accounted for in our prey predictions, because no literature was found reporting 1940 prey isotope values. As a result, our interpretation of E01 as not having fed in Antarctica during the earliest parts of its baleen may be erroneous (Fig 6, cm #0–20).

In summary, of all the individual baleen measurements, 72% were located within the isotopic ranges corresponding to an Antarctic, low-trophic level feeding signal, as expected from the classical feeding model (Fig 2, zone B). For D population individuals, 96% of samples were contained within this isotopic range, while only 59% of E1 samples correspond to classical feeding (S2 Fig). The supplementary feeding category corresponded to 21% of E1 samples and 4% of D samples that were outside the Antarctic range for δ^13^C and/or δ^15^N (S3 Fig). Whales identified as temperate zone feeders accounted for 20% of the E1 population observations, and were not found in D population individuals (S4 Fig).

**Fig 7:**
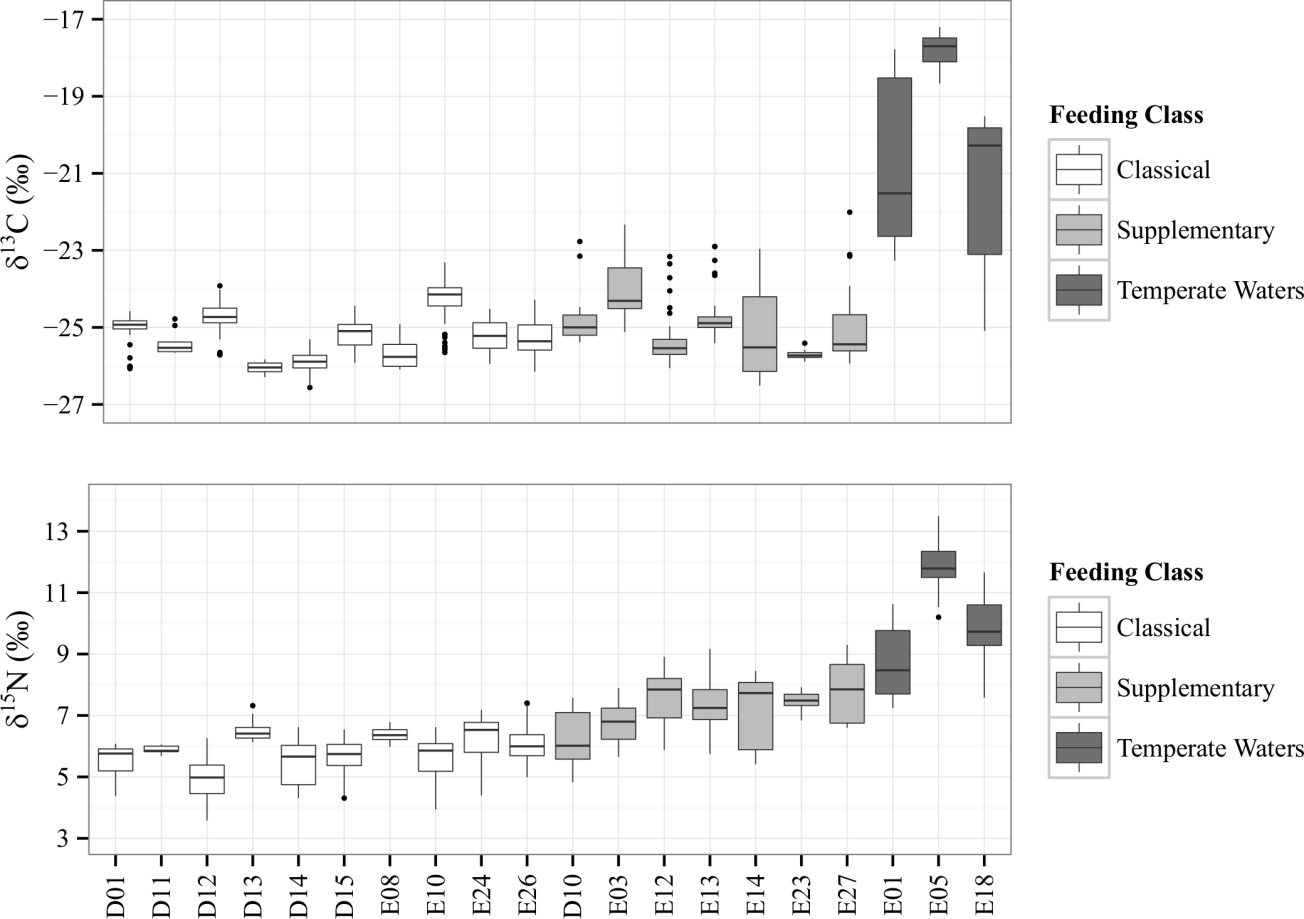
Boxplot representation of each individual whale according to feeding category. Representations of the spread of values for each individual baleen plate, with colours assigned as per feeding category. Boxes represent the 25^th^ and 75^th^ quartiles and are sectioned by the mean. Whiskers represent the 95^th^ quartiles, with outliers as dots.

The Kruskal-Wallis test determined that there were significant differences in variance and distribution between the three feeding categories and between the two populations for both δ^13^C and δ^15^N (p<0.05). Scheffe’s comparison of means between the different feeding groups showed that there were significant differences in δ^15^N between all categories but that temperate zone feeders were the only category significantly different in δ^13^C (Table 4, Fig 7).

**Table 4:** Scheffe comparison of means between the different feeding groups.

**δ**^**15**^**N**					
Category	N	Subset for alpha = 0.05			
		1	2	3	4
Classical D	184	5.4			
Classical E1	151		6.0		
Supplementary	189			7.1	
Temperate zone	93				10.2
Sig.		1.0	1.0	1.0	1.0
**δ**^**13**^**C**					
Category	N	Subset for alpha = 0.05			
		1		2	
Classical D	184	-25.2			
Classical E1	151	-24.9			
Supplementary	189	-24.9			
Temperate zone	93			-20.1	
Sig.		0.2		1.0	

Means for groups in homogeneous subsets are displayed. Groups were formed using the classification from Table 3.

This is consistent with the way the groups were formed, because we expect classical and supplementary feeders to be feeding primarily in Antarctica (with the same δ^13^C baseline) and temperate zone feeders to have consistently higher δ^13^C values from non-Antarctic feeding. The most unexpected result was that the classical feeding SHHW from the D and E1 populations had significantly different δ^15^N values (Table 4). The mean δ^15^N for classical feeders of both populations fit within our Antarctic krill feeding range, hence this observation may indicate some spatial δ^15^N variability in the average δ^15^N of Antarctic prey used by the different SHHW populations. Indeed, δ^15^N values lower than the defined Antarctic range were observed in individuals of both populations (D12 and E10, Table 3). Further work is needed to establish more detailed prey isoscapes for Antarctic waters.

The observation that six out of seven D population animals were assigned to the classical feeding category, compared to four out of thirteen E1 individuals (Table 3) could indicate that the D population is less likely to feed outside the traditional Antarctic zone than the E1 population. Whales from the E1 population may have greater opportunities to engage in supplementary feeding along the migration route when they pass productive waters around New-Zealand and Tasmania [6, 46, 77, 78]. Seamounts have been shown to be of potential importance for the New-Caledonia humpback whale breeding stock [79] and such interactions may exist in the E1 population. It is however acknowledged that stranded individuals are typically biased towards old, young or sick individuals, and hence both sample type and number preclude further discussion of ecological differences between the two breeding stocks.

## Conclusion

Stable isotope analysis of SHHW baleen plates from the two Australian breeding populations showed a greater diversity in feeding behaviour than expected. Evidence of feeding in temperate waters and partial migration was observed in half of the individuals studied, pointing to plasticity in both prey selection and migratory behaviour. This plasticity was more evident in the East coast population (nine out of thirteen E1 individuals) than in the West coast population (one out of seven D individuals). Further differences were observed between the classical feeders from the two populations, with significant differences in δ^15^N pointing at spatial variability between Antarctic feeding locations.

This study provides the first estimate of SHHW baleen elongation rate, which averaged 16 cm/year, and indication of a slowed elongation with age. Trophic fractionation in SHHWs corresponded to fin whale TFs reported by Borrell, Abad-Oliva [57] of +2.77‰ for δ^15^N and +2.26‰ for δ^13^C.

## Supporting Information

S1 Fig Isotopic profiles of paired plates from two individual whales. Two baleen plates were collected from two individual humpback whales. The resulting isotopic profiles were superposed to study reproducibility between plates from a single individual. Approximate migratory cycles are shown, with arrows indicating annual δ^15^N lows indicative of Antarctic feeding. Time flows left to right. D01: Plates A and B, E14: Plates C and D.
(TIFF)

S2 Fig Bivariate plot of classical feeders. Each zone is created using isotopic coordinates consistent with prey values and corrected for trophic fractionation. The whale isotope data plots within the zone appropriate to the prey consumed by the individual. Zones: Antarctic Krill (dashed line), Australian Krill (full line), and average Australian fish species (dotted line).
(TIFF)

S3 Fig Bivariate plot of supplementary feeders. Each zone is created using isotopic coordinates consistent with prey values and corrected for trophic fractionation. The whale isotope data plots within the zone appropriate to the prey consumed by the individual. Zones: Antarctic Krill (dashed line), Australian Krill (full line), and average Australian fish species (dotted line).
(TIFF)

S4 Fig Bivariate plot of temperate zone feeders. Each zone is created using isotopic coordinates consistent with prey values and corrected for trophic fractionation. The whale isotope data plots within the zone appropriate to the prey consumed by the individual. Zones: Antarctic Krill (dashed line), Australian Krill (full line), and average Australian fish species (dotted line).
(TIFF)

S1 Table Extended sample details. “MN” is the shorthand for *Megaptera novaeanglia*, “STRND” for stranded and “EUT” for euthanized. “E1”/”E” and “D” are the respective populations. “QLD”, “WA”, “VIC”, “NSW”, “TAS” and “SA” respectively refer to Queensland, Western Australia, Victoria, New South Wales, Tasmania and South Australia. ^1^Samples from museum collections have an additional ID number reflecting the museum’s collection ID. All collections are Australian collections. ^2^Museum Victoria, Carlton VIC. ^3^Australian Museum, Sydney NSW. ^4^Southern Ocean Persistent Organic Pollutant Program (SOPOPP), Brisbane QLD. ^5^Tasmanian Department of Primary Industries, Parks, Water and Environment (DPIPWE), TAS. ^6^South Australian Museum, Adelaide SA. ^7^Ceduna National Trust Museum, Ceduna SA. ^8^Care of Dr Carlysle Holyoake, Murdoch University WA.
(DOCX)

S2 Table Expected isotopic values for prey items. Average isotopic values (with standard deviation) for prey items liable to be consumed by Australian humpback whale populations. These have not been adjusted for trophic level.
(DOCX)
