# Supplementary figures and images for: Isotopic Evidence of a Wide Spectrum of Feeding Strategies in Southern Hemisphere Humpback Whale Baleen Records
Source: PLoS One. 2016 May 31;11(5):e0156698. doi: 10.1371/journal.pone.0156698 (PMC4887117; doi:10.1371/journal.pone.0156698)

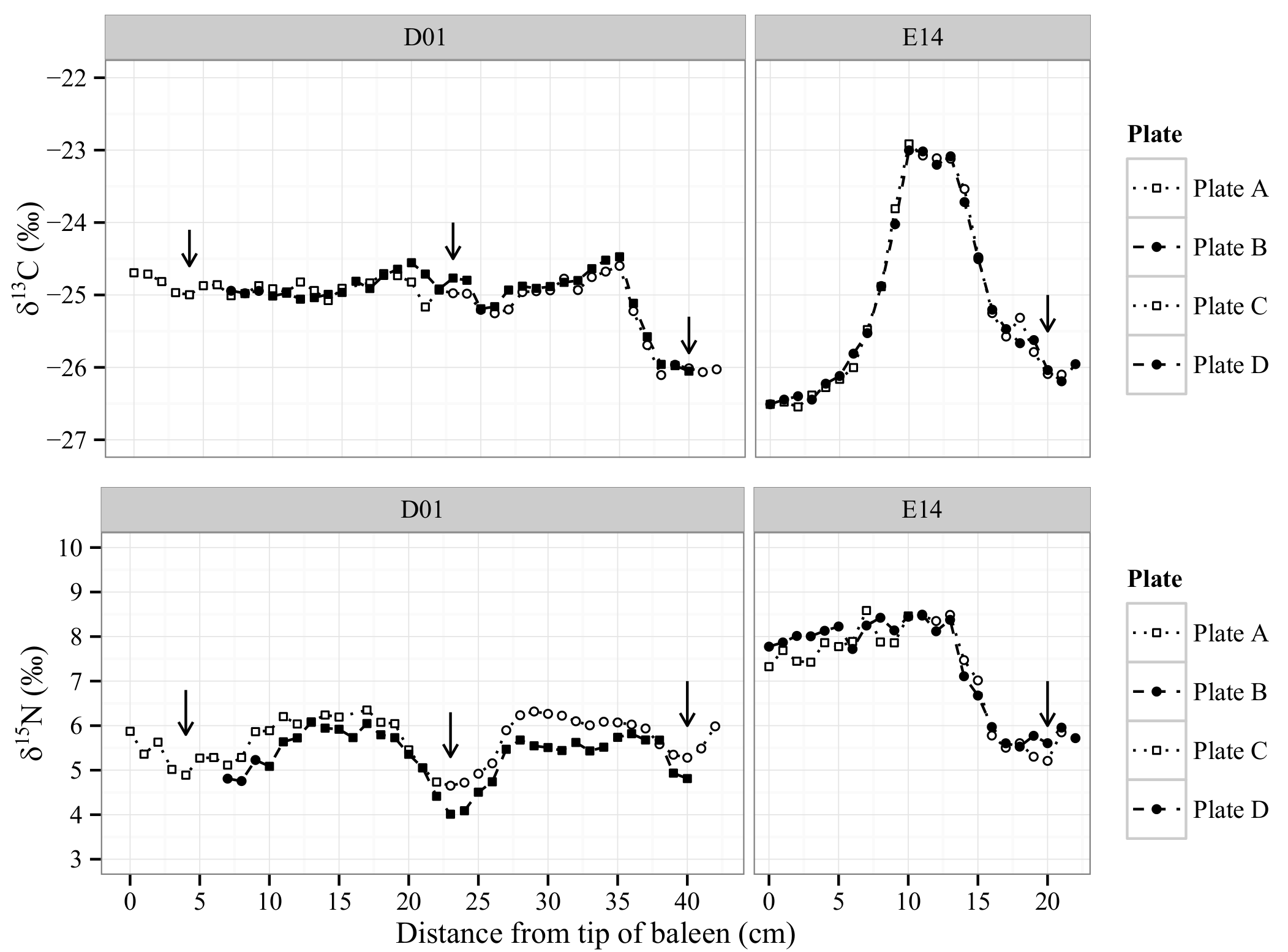

Supplement: S1 Fig — Two baleen plates were collected from two individual humpback whales. The resulting isotopic profiles were superposed to study reproducibility between plates from a single individual. Approximate migratory cycles are shown, with arrows indicating annual δ15N lows indicative of Antarctic feeding. Time flows left to right. D01: Plates A and B, E14: Plates C and D. (TIFF) [file pone.0156698.s001.tiff]

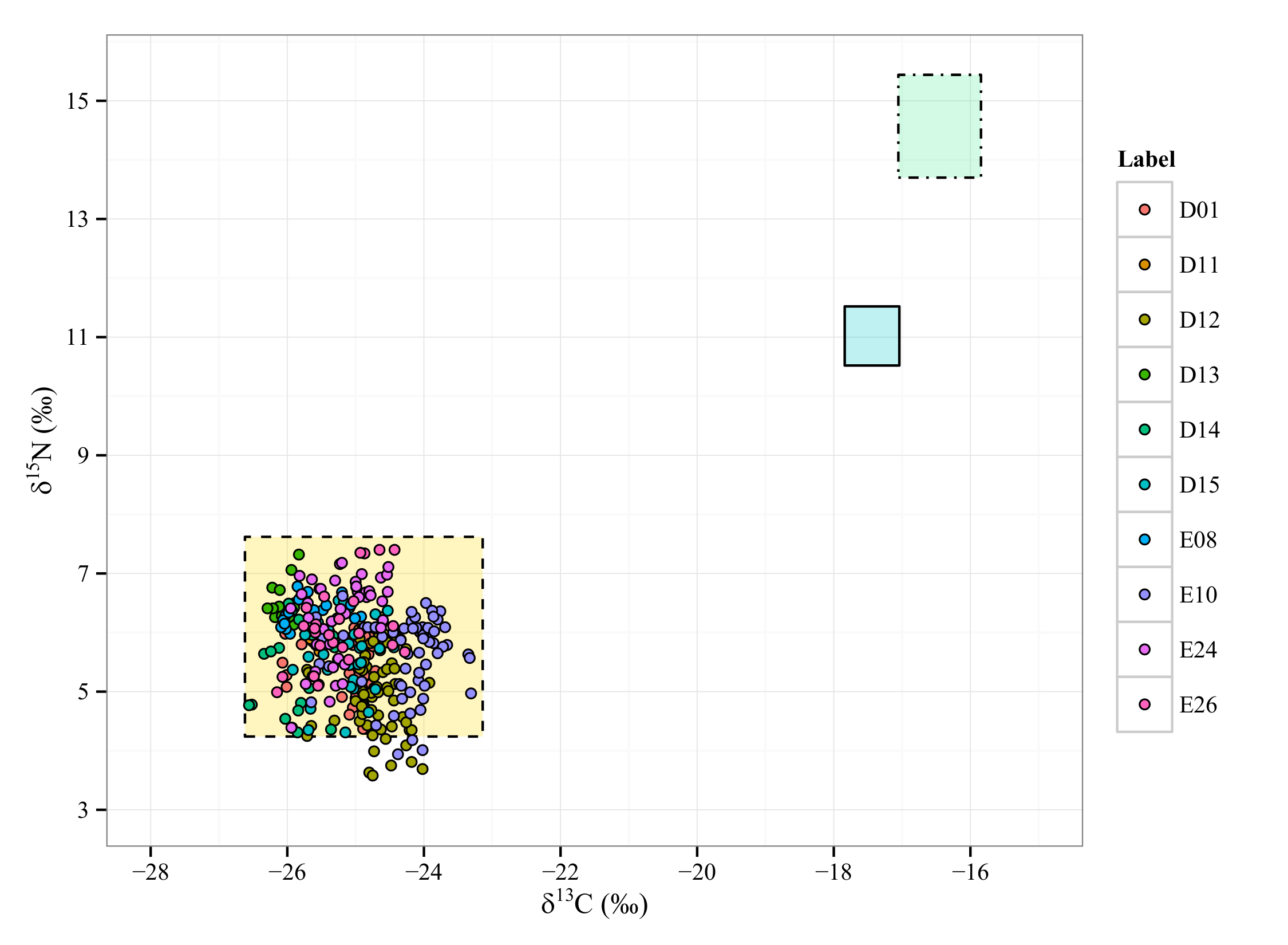

Supplement: S2 Fig — Each zone is created using isotopic coordinates consistent with prey values and corrected for trophic fractionation. The whale isotope data plots within the zone appropriate to the prey consumed by the individual. Zones: Antarctic Krill (dashed line), Australian Krill (full line), and average Australian fish species (dotted line). (TIFF) [file pone.0156698.s002.tiff]

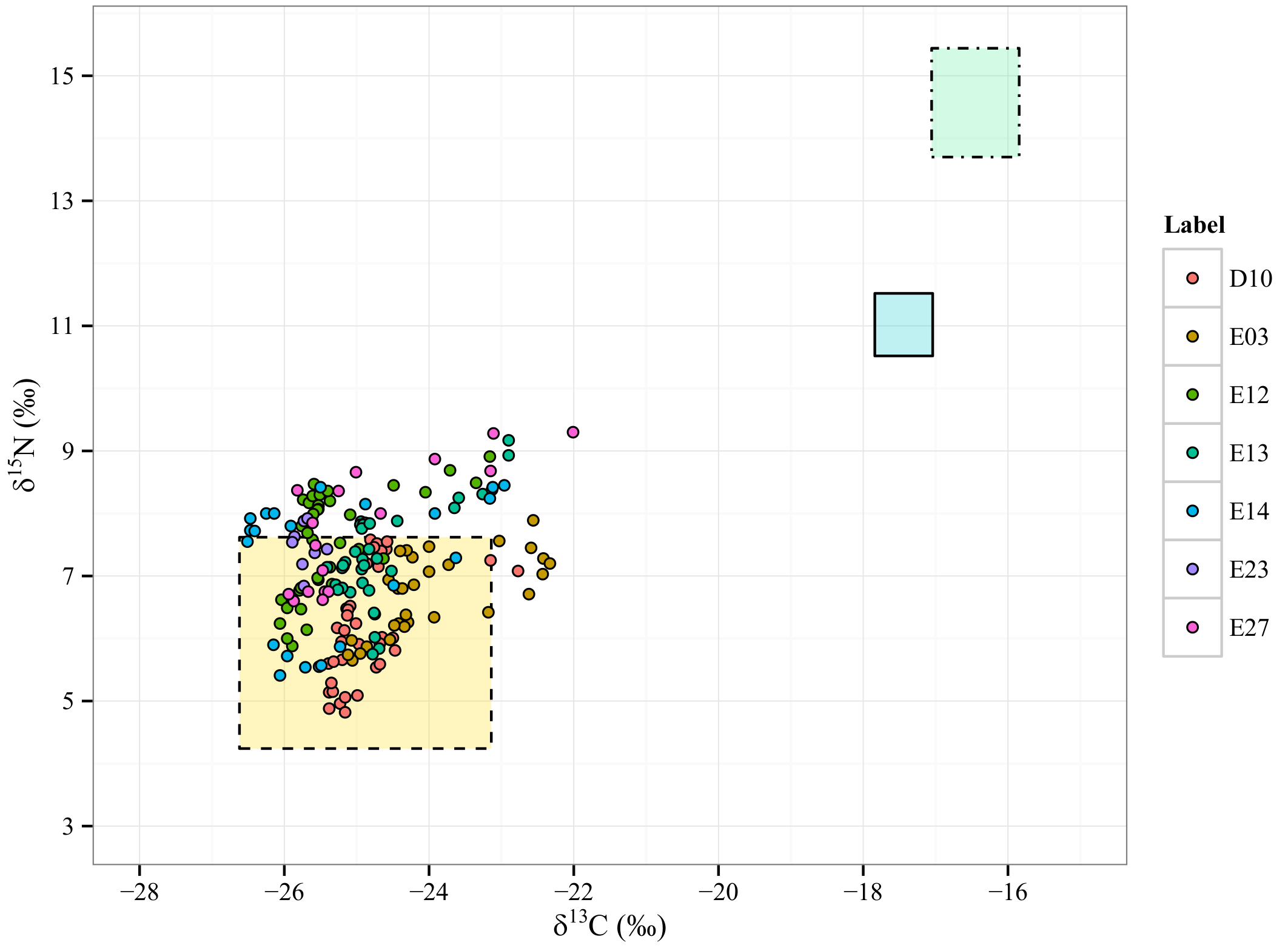

Supplement: S3 Fig — Each zone is created using isotopic coordinates consistent with prey values and corrected for trophic fractionation. The whale isotope data plots within the zone appropriate to the prey consumed by the individual. Zones: Antarctic Krill (dashed line), Australian Krill (full line), and average Australian fish species (dotted line). (TIFF) [file pone.0156698.s003.tiff]

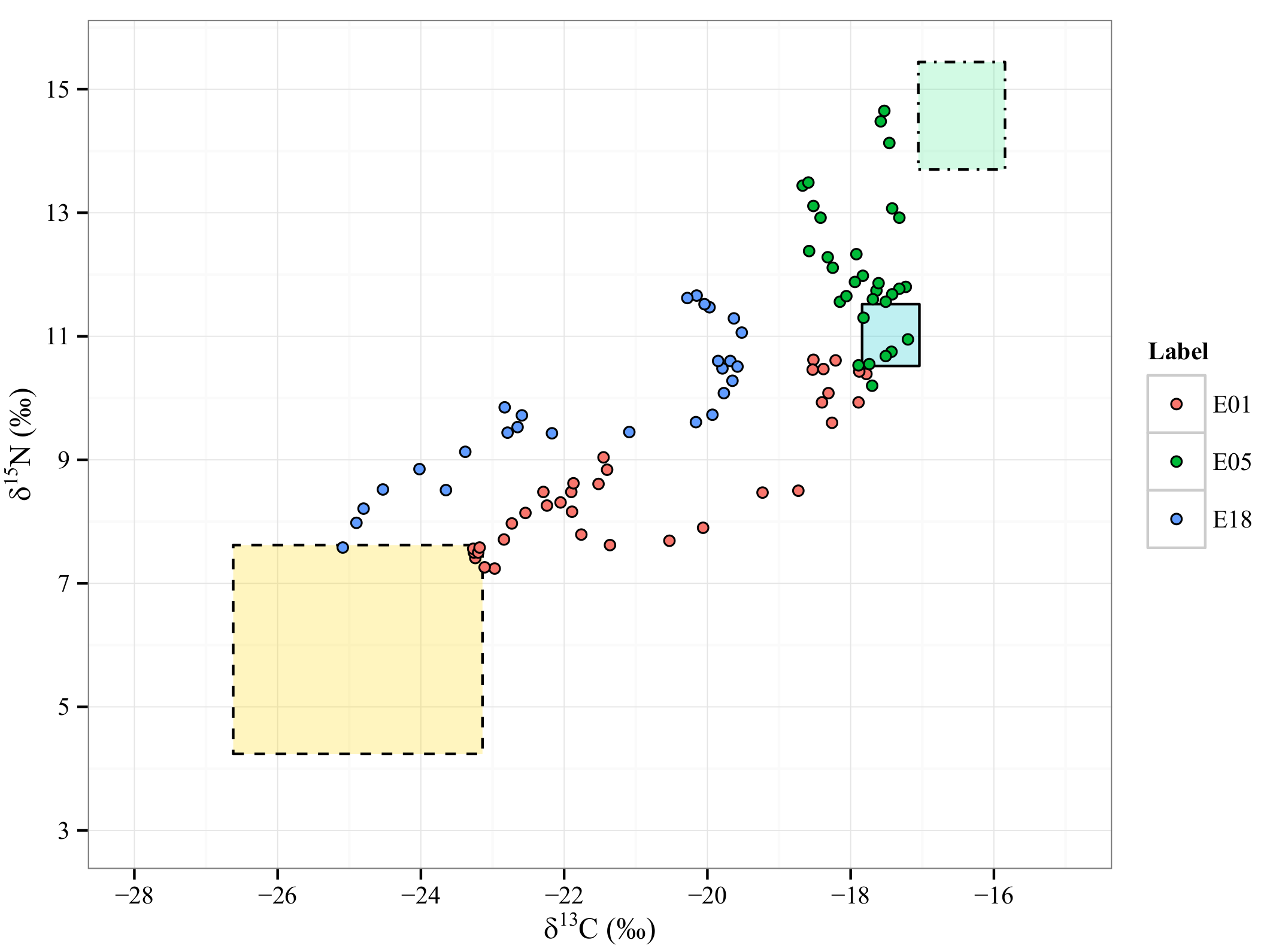

Supplement: S4 Fig — Each zone is created using isotopic coordinates consistent with prey values and corrected for trophic fractionation. The whale isotope data plots within the zone appropriate to the prey consumed by the individual. Zones: Antarctic Krill (dashed line), Australian Krill (full line), and average Australian fish species (dotted line). (TIFF) [file pone.0156698.s004.tiff]
