# Supplementary material for: Isotopic Evidence of a Wide Spectrum of Feeding Strategies in Southern Hemisphere Humpback Whale Baleen Records
Source: PLoS One. 2016 May 31;11(5):e0156698. doi: 10.1371/journal.pone.0156698 (PMC4887117; doi:10.1371/journal.pone.0156698)
Supplement: S1 Table — “MN” is the shorthand for Megaptera novaeanglia, “STRND” for stranded and “EUT” for euthanized. “E1”/”E” and “D” are the respective populations. “QLD”, “WA”, “VIC”, “NSW”, “TAS” and “SA” respectively refer to Queensland, Western Australia, Victoria, New South Wales, Tasmania and South Australia. 1Samples from museum collections have an additional ID number reflecting the museum’s collection ID. All collections are Australian collections. 2Museum Victoria, Carlton VIC. 3Australian Museum, Sydney NSW. 4Southern Ocean Persistent Organic Pollutant Program (SOPOPP), Brisbane QLD. 5Tasmanian Department of Primary Industries, Parks, Water and Environment (DPIPWE), TAS. 6South Australian Museum, Adelaide SA. 7Ceduna National Trust Museum, Ceduna SA. 8Care of Dr Carlysle Holyoake, Murdoch University WA. (DOCX) [file pone.0156698.s005.docx]

Table S1: Extended samples details.

| **Name** | **Internal Laboratory ID**  **(Museum ID)^1^** | **Stranding location** | **Collector**^1^ |
| --- | --- | --- | --- |
| E01 | MN.E1.STRND.01.40  (#C24961) | Cape Woolamai, Philip Island (VIC) | Museum Victoria^2^ |
| E03 | MN.E1.STRND.02.89  (#C29136) | Browns Rock, Fraser Island (QLD) | Museum Victoria^2^ |
| E05 | MN.E1.STRND.01.98  (#M35090) | Woolgoolga - Safety Beach (NSW) | Australian Museum^3^ |
| E08 | MN.E1.STRND.01.10 | Tallebudgera (QLD) | SOPOPP^4^ |
| E10 | MN.E1.STRND.03.11 | Main Beach, North Stradbroke Is. (QLD) | SOPOPP^4^ |
| E12 | MN.E1.STRND.09.11 | Tasmanian Peninsula (TAS) | DPIPWE^5^ |
| E13 | MN.E1.STRND.10.11 | Bruny Island (TAS) | DPIPWE^5^ |
| E14 | MN.E1.STRND.02.12 | Fraser Is., South Eli beach (QLD) | SOPOPP^4^ |
| E18 | MN.E1.STRND.07.12  (#C37108.1) | Venus Bay (VIC) | Museum Victoria^2^ |
| E23 | MN.E1.STRND.01.14 | Moolloolaba (QLD) | SOPOPP^4^ |
| E24 | MN.E1.STRND.03.10  (#M25695) | Franklin Is. (SA) | South Australian Museum^6^ |
| E26 | MN.E1.STRND.05.10  (N/A) | Rocky Point (SA) | Ceduna National Trust Museum^7^ |
| E27 | MN.E1.STRND.03.89 (#M15187) | Investigator Strait, Yorke Peninsula (SA) | South Australian Museum^6^ |
| D01 | MN.D.STRND.01.07 | Hillary's Boat Harbour (WA) | Murdoch University^8^ |
| D10 | MN.D.STRND.03.13 | Whitfords beach (WA) | Murdoch University^8^ |
| D11 | MN.D.STRND.04.13 | North Hillarys Boat Harbour (WA) | Murdoch University^8^ |
| D12 | MN.D.STRND.05.13 | (WA) | Murdoch University^8^ |
| D13 | MN.D.STRND.01.14 | (WA) | Murdoch University^8^ |
| D14 | MN.D.STRND.03.14 | (WA) | Murdoch University^8^ |
| D15 | MN.D.STRND.04.14 | (WA) | Murdoch University^8^ |

“MN” is the shorthand for *Megaptera novaeanglia*, “STRND” for stranded and “EUT” for euthanized. “E1”/”E” and “D” are the respective populations. “QLD”, “WA”, “VIC”, “NSW”, “TAS” and “SA” respectively refer to Queensland, Western Australia, Victoria, New South Wales, Tasmania and South Australia.

^1^Samples from museum collections have an additional ID number reflecting the museum’s collection ID. All collections are Australian collections.

^2^Museum Victoria, Carlton VIC. ^3^Australian Museum, Sydney NSW. ^4^Southern Ocean Persistent Organic Pollutant Program (SOPOPP), Brisbane QLD. ^5^Tasmanian Department of Primary Industries, Parks, Water and Environment (DPIPWE), TAS. ^6^South Australian Museum, Adelaide SA. ^7^Ceduna National Trust Museum, Ceduna SA. ^8^Care of Dr Carlysle Holyoake, Murdoch University WA.
