# Supplementary material for: Isotopic Evidence of a Wide Spectrum of Feeding Strategies in Southern Hemisphere Humpback Whale Baleen Records
Source: PLoS One. 2016 May 31;11(5):e0156698. doi: 10.1371/journal.pone.0156698 (PMC4887117; doi:10.1371/journal.pone.0156698)
Supplement: S2 Table — Average isotopic values (with standard deviation) for prey items liable to be consumed by Australian humpback whale populations. These have not been adjusted for trophic level. (DOCX) [file pone.0156698.s006.docx]

Table S2: Expected isotopic values for baleen plates.

|  | **δ^15^N** | | **δ^13^C** | |
| --- | --- | --- | --- | --- |
|  | **Generic TF: +3.4‰** | **Fin Whale TF: +2.77‰** | **Generic TF: +0.5‰** | **Fin Whale TF:**  **+2.26‰** |
| **Adult whales on 100% Antarctic Krill** | 6.56 | 5.93 | -26.64 | -24.88 |
| **Adult whales on 100% Australian Krill** | 11.65 | 11.02 | -20.3 | -17.74 |
| **Adult whales on 100% fish (averaged sources)** | 15.19 | 14.56 | -18.21 | -16.45 |

Average isotopic values (with standard deviation) for prey items liable to be consumed by Australian humpback whale populations. These have not been adjusted for trophic level.
